# The voices of medical education scholarship: Describing the published landscape

**DOI:** 10.1111/medu.14959

**Published:** 2022-11-08

**Authors:** Lauren A. Maggio, Joseph A. Costello, Anton Boudreau Ninkov, Jason R. Frank, Anthony R. Artino

**Affiliations:** ^1^ Medicine Uniformed Services University of the Health Sciences Bethesda Maryland USA; ^2^ Center for Health Professions Education, Henry M. Jackson Foundation Bethesda Maryland USA; ^3^ École de bibliothéconomie et des sciences de l'information Université de Montréal Montréal Québec Canada; ^4^ Department of Emergency Medicine University of Ottawa Ottawa Ontario Canada; ^5^ George Washington University School of Medicine and Health Sciences Washington District of Columbia USA

## Abstract

**Introduction:**

The voices of authors who publish medical education literature have a powerful impact on the field's discourses. Researchers have identified a lack of author diversity, which suggests potential epistemic injustice. This study investigates author characteristics to provide an evidence‐based starting point for communal discussion with the intent to move medical education towards a future that holds space for, and values, diverse ways of knowing.

**Method:**

The authors conducted a bibliometric analysis of all articles published in 24 medical education journals published between 2000 and 2020 to identify author characteristics, with an emphasis on author gender and geographic location and their intersection. Article metadata was downloaded from Web of Science. Genderize.io was used to predict author gender.

**Results:**

The journals published 37 263 articles authored by 62 708 unique authors. Males were more prevalent across all authorship positions (*n =* 62 828; 55.7%) than females (*n =* 49 975; 44.3%). Authors listed affiliations in 146 countries of which 95 were classified as Global South. Few articles were written by multinational teams (*n =* 3765; 16.2%). Global South authors accounted for 12 007 (11.4%) author positions of which 3594 (3.8%) were female.

**Discussion:**

This study provides an evidence‐based starting point to discuss the imbalance of author voices in medical education, especially when considering the intersection of gender and geographical location, which further suggests epistemic injustice in medical education. If the field values a diversity of perspectives, there is considerable opportunity for improvement by engaging the community in discussions about what knowledge matters in medical education, the role of journals in promoting diversity, how to best use this baseline data and how to continue studying epistemic injustice in medical education.

## Abstract 2

Authorship counts reveal an imbalance of voices in #meded, especially when considering the intersection of gender & geography, thereby providing a starting point for discussion of how to move towards a field that values diverse ways of knowing.

## 1 INTRODUCTION

Academic publishing is the primary means of disseminating scientific knowledge in medical education. Thus, the voices of the researchers who author those publications are the voices that are heard. Ultimately, they are the voices that influence what is ‘known’ in the field. However, recent research suggests that author diversity across scientific disciplines, as measured by gender and race,^1^, ^2^ as well as international representation, as measured by country of institutional affiliation,^3^, ^4^ are lacking. Research in medical education has drawn similar conclusions, although the scope of that work has been fairly narrow.^4^, ^5^, ^6^, ^7^, ^8^ Taken together, this lack of diversity suggests epistemic injustice, which has implications for who has a voice in medical education and who is excluded from the conversation.

Epistemic injustice is injustice pertaining to knowledge or epistemic goods (e.g. information or education).^9^ When Fricker coined the term in 2007, she chose the label to ‘delineate a distinctive class of wrongs, namely those in which someone is ingenuously downgraded or disadvantaged in respect to their status as an epistemic subject’.^10^ ^(p54)^ Fricker described two related forms of epistemic injustice: testimonial and hermeneutical. Testimonial injustice occurs interpersonally when the receiver of knowledge allows an individual or group's knowledge to be discredited (i.e. considered epistemically lesser) based on race, gender, profession, geographical location and so on.^10^ An example would be a peer reviewer who provides a ‘deflated level of credibility’^9^ ^(p1)^ to an author's findings because the author is not a native English speaker.

Hermeneutical injustice occurs at the systems level and is defined as ‘the injustice of having some significant area of one's social experience obscured from collective understanding owing to a structural identity prejudice in the collective hermeneutical resource’.^9^ ^(p155)^ To build on the previous example, hermeneutical injustice would be the systematic exclusion of research by non‐English speaking authors due to the dominant culture not accepting their work as credible or legitimate.

As these examples demonstrate, epistemic injustice has implications for what is included in the collective knowledge base, which can lead to systematic underrepresentation or silencing of marginalised and minoritized groups.^11^, ^12^ This underrepresentation exacerbates existing injustices and inequalities.^9^ It also unfairly advantages those individuals whose experiences are represented, such that their voices are likely overrepresented. This results in a set of ‘collective social understandings’ and perceived truths of a field, whether or not those understandings and truths are truly representative.^9^ ^(p147)^

In 2022, Kursurkar invoked the metaphor of the leaky pipeline to describe knowledge generation in medical education: specifically, how knowledge from the Global South is ‘lost and never reaches or is never incorporated into the Global North medical education research reservoir’.^13^ In making this statement, she draws upon personal experience and points to research in medical education that has examined small slices of the literature using bibliometric methods.^4^, ^7^, ^14^, ^15^, ^16^, ^17^ For example, in 2013, Lee and colleagues^14^ investigated the evolution of medical education publications over a 50‐year period; these publications were identified as ‘medical education’ based on search terms. Three years later, Azer produced a ranking of medical education articles based on citations from 13 journals.^15^ More recently, Madden et al. described the gender of authors and editors in four medical education journals,^7^ and Maggio et al. investigated author gender and geographic and institutional affiliation in a knowledge syntheses.^4^ These studies each provide a valuable glimpse into the field's evidence base, suggesting that—based on their specific and somewhat narrow sampling techniques—medical education is a growing field with much of its research being conducted in North American and European contexts and increasingly by women. However, we are unaware of any research in medical education that provides a current, comprehensive overview of author characteristics or one that examines the intersection of characteristics such as gender and geographic affiliation. Without this knowledge, it is difficult to understand the presence or magnitude of epistemic injustice in medical education.

Unfortunately, identifying and studying claims of epistemic injustice are not straightforward endeavours,^11^ because it is difficult to directly examine that which is absent. To do so, we must have a starting point or baseline. In this study, we provide that starting point by describing the authorship landscape of the last 20 years of medical education literature, using the largest database of scholarship assembled to date. Our intent is to identify those voices present in the dominant scholarship (i.e. those that are ‘epistemically advantaged’ and published in journals explicitly recognised as medical education focused) to provide an evidence base for new and ongoing global conversations about whose voices are missing. We recognise that this approach runs the risk of perpetuating existing patterns and inequalities by highlighting dominant power structures. However, our sincere hope is to raise awareness about epistemic injustice and begin moving medical education towards a more just future that holds space for, and values, diverse ways of knowing.^18^

## 2 METHODS

We conducted a comprehensive bibliometric analysis of all articles published in 24 medical education journals published between 2000 and 2020 with a focus on authors' gender and geographical affiliation. We chose to focus on these two author characteristics to build on the existing, narrowly focused literature^1^, ^2^, ^3^, ^4^, ^7^, ^16^, ^17^, ^19^, ^20^, ^21^ and to extend the conversation by understanding the intersection of these two characteristics, which has not been previously explored in medical education but has shown to be an issue in other disciplines.^22^, ^23^

Our sample consisted of articles published between 2000 and 2020 in the 24 journals featured on the MEJ‐24 (See Appendix S1 for journal listing).^24^ The MEJ‐24 has been proposed as ‘a seed set of journals’ that constitutes the field of medical education and was derived using the evidence‐based approach of journal co‐citation. Journal co‐citation is a bibliometric technique utilised by researchers to describe a field based on the relationships between journals and their citations.^25^, ^26^, ^27^ For these 24 journals, on 27 August 2021, we retrieved metadata for 22 of these journals from the database Web of Science (WoS). On the same day, for the remaining two journals not indexed in WoS (*Journal of Graduate Medical Education (JGME)* and *The Canadian Medical Education Journal (CMEJ)*), we downloaded article metadata from the Crossref REST API. For all 24 journals, we downloaded the following data for the present study: journal name, author names and affiliations and publication date. We selected WoS based on its well‐defined metadata and long history as a valuable tool for bibliometric analyses across multiple disciplines.^28^ For the 22 journals indexed in WoS, we also downloaded the number of times an article was cited, its cited references, open access status and the references of any articles that had cited it. These citation data were unavailable for *JGME* and *CMEJ*. For all journals, we retrieved the journal scope note directly from their website. All metadata was organised in an Excel spreadsheet.^29^

From each article, we extracted the names of all authors. However, in order to accurately analyse author‐level data, it was essential to adequately disambiguate author names that represent the same (or different) people.^30^ Thus, we created a thesaurus of author names to reconcile name permutations (e.g. Artino, AR, Jr and Artino, Anthony R, Jr were both reconciled to Artino, Anthony). Complete details of author name disambiguation and the full author thesaurus are freely accessible on Zenodo, an open source research data respository.^31^ Once the author data were cleaned, to make a prediction of the authors' gender using their first name, we utilised the tool Genderize.io.^32^ We recognise that our effort to predict gender is an oversimplification of a complex social construct, especially because an individual's gender is best described by that individual, and our method did not allow us to capture authors who identify as non‐binary. However, currently, there does not exist a resource that provides gender information for individual authors. Moreover, multiple publications with similar goals to this current study have utilised Genderize.io as a gender prediction tool.^33^, ^34^, ^35^, ^36^ Therefore, we relied on the Genderize.io tool, in keeping with work done previously by authors using bibliometric methods.^4^, ^17^, ^33^, ^34^, ^35^, ^36^, ^37^ Genderize.io bases its predictions on a database of over 100 million names and for each name provides the “percent confidence” that the gender prediction is correct. We accepted the tool's gender prediction if and when it was over 70% confident of the gender prediction. To our knowledge, there is no standard cutoff for gender prediction accuracy. Thus, we selected 70% confidence based on our review of studies employing Genderize.io that used thresholds ranging from 60%–90%.^34^, ^37^

For all articles, we identified the country for each author based on the location of their institutional affiliation. To identify these affiliations, we included any article with at least one author–institution affiliation. As institutional affiliations are often variably reported in published papers, we created an additional thesaurus of affiliations, which enabled us to accommodate these variations (e.g. Univ Nebraska and Nebraska Univ reconciled to University of Nebraska) and to associate institutions (e.g. university hospitals) with a parent institution. The thesaurus and full details of its creation are available on Zenodo.^31^ Additionally, we identified countries as being in the ‘Global South or North’, which is a designation that relies on a geographical approach taking i account a country's gross domestic product, income hierarchies, economic growth rates, foreign policy adherence and political decisions.^38^, ^39^, ^40^ We recognise that the terminology of the Global South and North is not clear cut^40^, ^41^, ^42^; however, we feel that it provides a feasible starting point for this investigation.

We used Excel^29^ to calculate descriptive statistics.

## 3 RESULTS

Across the MEJ‐24, 37 263 articles were published. Of these articles, 47 listed no author and 212 were attributed to an anonymous author. We excluded these articles (0.7%) from our analysis given our emphasis on authorship characteristics and to facilitate consistency across analyses. Of the remaining 37 004 articles, the largest number of articles were published in 2020 (*n =* 3957, 10.7%) and the smallest number in 2000 (*n =* 711, 1.9%). This difference in articles published represents a 456.5% increase over the time period examined. *Academic Medicine* (*n =* 7760, 21%), *Medical Education* (*n =* 5499, 14.9%) and *Medical Teacher* (*n =* 5029, 13.6%) published the most articles, accounting for 49.5% of all publications. Thirteen of the included journals indicated that they had an international or worldwide focus and/or reach.

Articles published in the 22 journals with citation data (*n =* 34 652) were cited 548 358 times. On average, articles were cited 15.8 times (stdev = 45.54 Range:0–3310). There were 6464 (18.7%) articles with no citations, of which 1333 were published in 2020. ‘Making sense of Cronbach's alpha’ published by the *International Journal of Medical Education* in 2011 was the most cited article in the dataset (*n =* 3310) (see Table S1 for a listing of top cited authors).^43^ Nearly half of the articles published in all 24 journals (*n =* 16 817; 45.5%) were openly accessible. For those that were openly accessible and that also had citation data (*n =* 34 652), the average citation rate was 15.0, which was just below the overall average citation rate.

### 3.1 Authors

We identified 139 325 authorship positions. The average author team included 3.8 (range 1–80; median = 3, stdev = 2.6) authors. If we exclude all single‐authored papers (*n =* 6884, 18.6%), the average team size increases to 4.4 members. The largest team included 80 authors who conducted a non‐randomised, multicenter trial on anti‐stigma training (towards patients with mental illness) for medical students.^44^ In 2000, the average team size was 2.7 (range: 2–14; median: 2) authors, and in 2020, the average team size was 4.3 (range: 2–40; median: 4), which represents a 57.3% increase in average author team size.

By disambiguating author names, we identified 62 078 unique authors representing 14 573 unique first names. For these names, we excluded unknown names (*n =* 1473) and any names with less than a 70% probability of a gender match (male [*n =* 675], female [*n =* 636]) as predicted by Genderize.io. This resulted in 5418 female and 6532 male first names used for our analysis.

**FIGURE 1:**
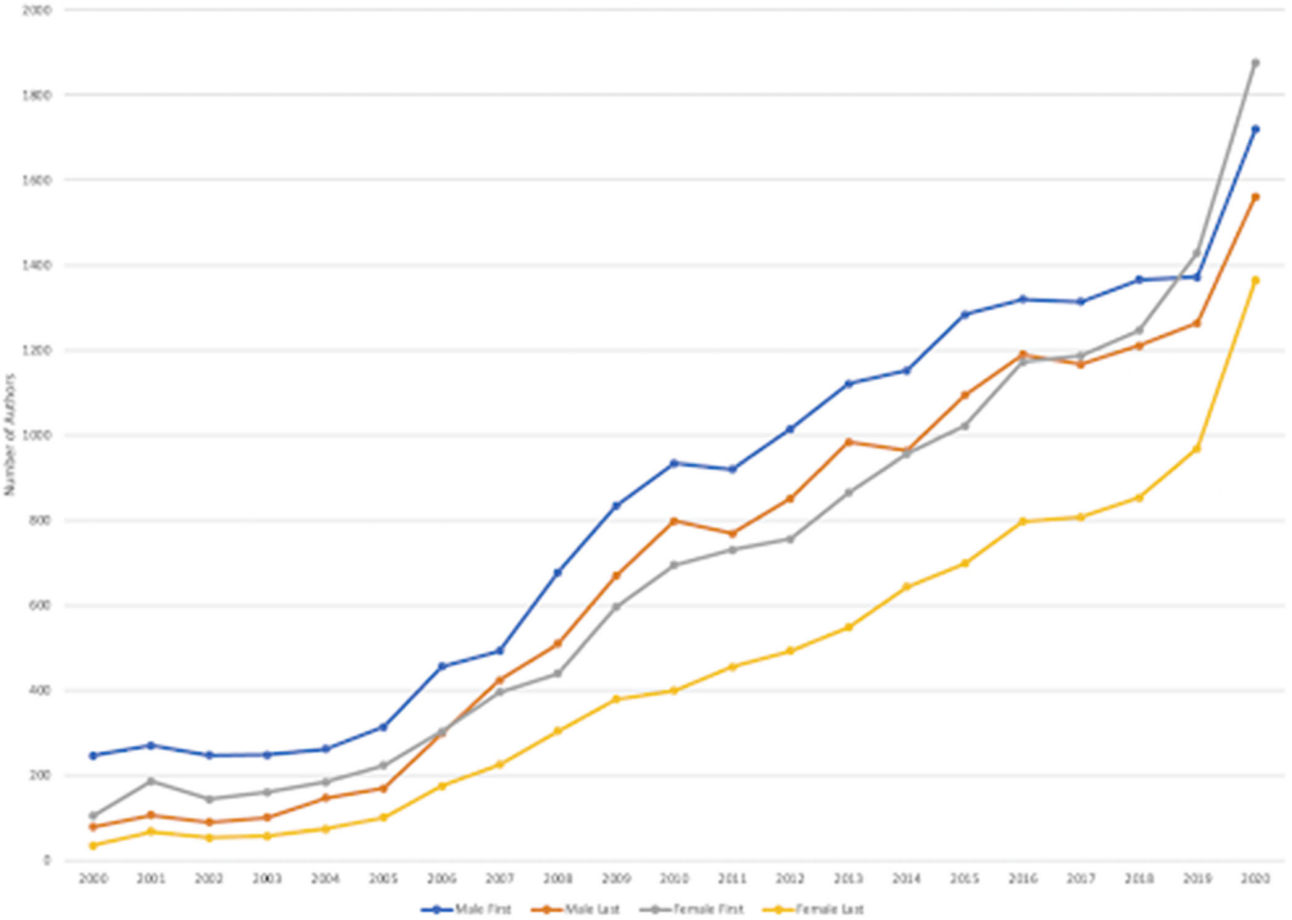
A comparison over time of male and female first and last authors publishing multi‐author articles in medical education journals between 2000 and 2020 [Color figure can be viewed at wileyonlinelibrary.com]

Applied to our unique author list, Genderize.io predicted 62 828 males (55.7%) and 49 975 females (44.3%) total. On multi‐author teams, 51.7% of first authors and 60.4% of last authors were males (See Table 1). In addition, males wrote 57.1% of single‐authored articles, of which 45% were categorised by WoS as editorials. Multi‐authored articles with female first authors (*n =* 12 739) were cited on average 16.3 times (37.7 = stdev) in comparison with 17.7 average (54.4 = stdev) for males (*n =* 13 632). Single author citation averages for males and females were 10.4 (34.2 = stdev) and 8.6 (40.4 = stdev), respectively. Figure 1 provides a comparison of author gender over the 21‐year time period for first and last authors on multi‐author articles.

**TABLE 1:** Predicted author gender for articles published in 22 medical education journals published between 2000 and 2020

	Male (%)	Female (%)
All author positions	62 828 (55.7)	49 975 (44.3)
Single author	3932 (67.1)	1925 (32.9)
First author (team)	13 632 (51.7)	12 739 (48.3)
Last author (team)	15 807 (60.4)	10 376 (39.6)
Not first or last author (team)	33 389 (42.2)	26 860 (34.0)

*Note*: We were unable to predict the gender of 3964 authors (Genderize.io returned: ‘unknown’ [*n =* 1325], male under 0.70 probability (*n =* 1406), female under 0.70 probability [*n =* 1233]).

### 3.2 Geographic affiliation

Due to metadata limitations, we included 28 805 articles with affiliation data. Authors listed affiliations in 146 of 195^45^ (74.9%) countries worldwide of which 95 were classified as Global South. There were 3751 (13.0%) authors affiliated with Global South institutions.

Overall, the United States (US) (42 236 authors, 40.4%), United Kingdom (UK) (12 967 authors, 12.4%) and Canada (10 505 authors, 10.0%), all of which are considered to be in the Global North, were the most represented countries, with 69.5% of all articles including at least one author from these three nations. In the Global South, the top three countries accounted for 4382 authors (4.2%) and included the following: China (1786 authors, 1.7%), South Africa (1358 authors, 1.3%) and Saudi Arabia (1238 authors, 1.2%) (See Table 2 for Top 10 geographic affiliations).

**TABLE 2:** Top 10 geographic affiliations reported in articles in medical education journals published between 2000 and 2020

Country	Overall appearances of an affiliation	First author (team)	Last author (team)	Single authors
United States (GN)	42 236	8771	8377	2466
United Kingdom (GN)	12 967	3263	3095	1211
Canada (GN)	10 505	2360	2212	535
Australia (GN)	5774	1373	1348	271
Netherlands (GN)	5304	1009	1211	154
Germany (GN)	4220	803	777	41
China (GS)	1786	326	238	29
South Africa (GS)	1358	374	228	97
Saudi Arabia (GS)	1238	272	156	62
Japan (GN)	1013	172	159	12

Abbreviations: GN, Global North; GS, Global South.

For multi‐authored articles (*n =* 23 257), a minority of articles were written by multinational teams (*n =* 3765; 16.2%). For the 5617 solo authored articles, authors represented 88 of 195 countries (45.1%), with 528 (9.4%) classified as countries in the Global South (See Table 2).

Authors in the Global South accounted for 12 007 (11.4%) author positions. There were 2187 authors teams with all members with Global South affiliations. For the first author position, 2120 authors (7.9%) were affiliated with institutions in the Global South.

**FIGURE 2:**
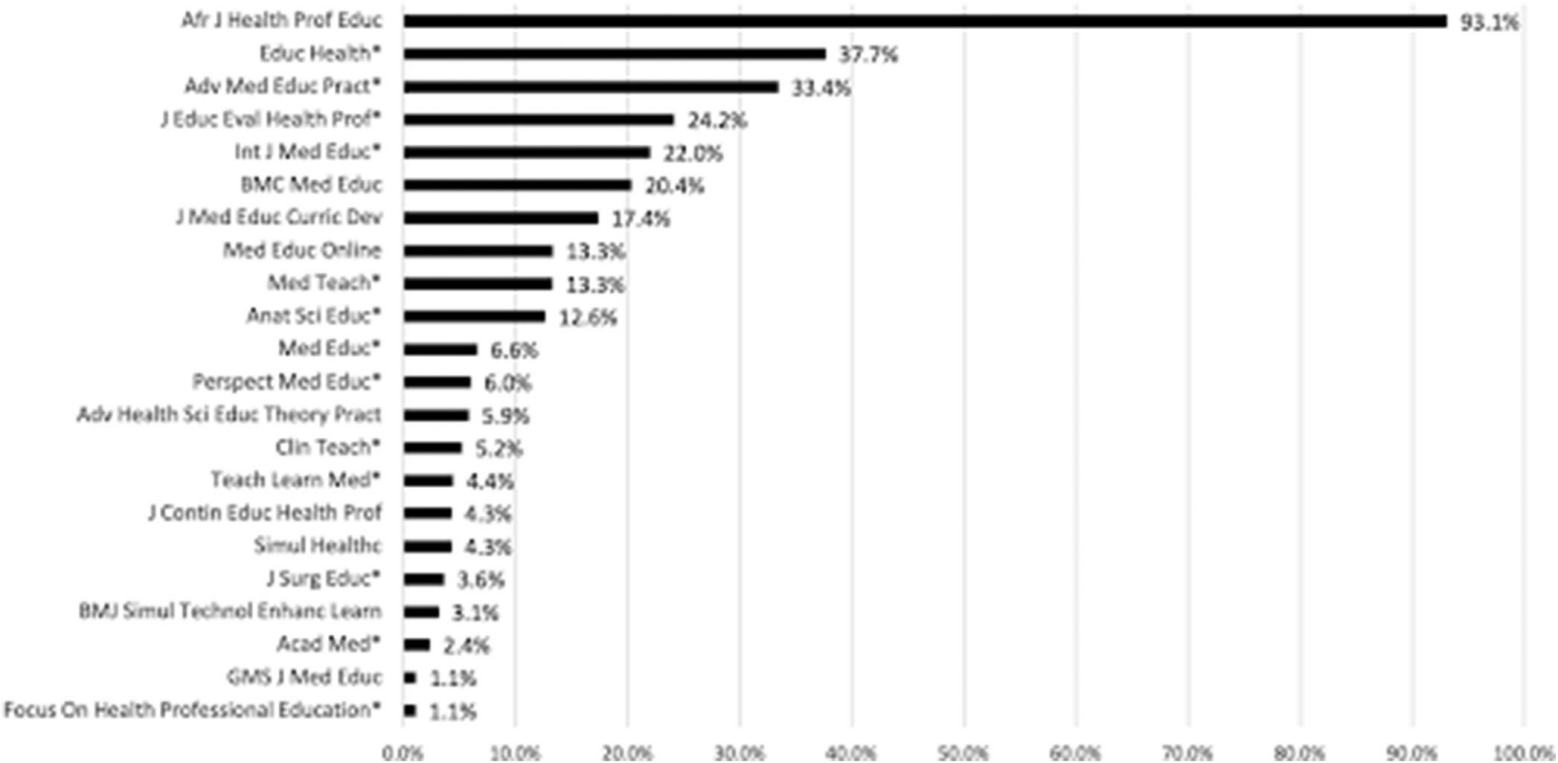
Journals listed by percentage of articles featuring at least one author from the Global South. *The journal's scope note indicates that the journal has an international scope.

All included journals published a mix of authors from the Global South and Global North to varying degrees. Figure 2 displays for each journal the percentage of articles that include at least a single Global South author. The scope notes for the majority journals (*n =* 13; see Figure 2) indicated that the journal had an international scope. Overall *BMC Medical Education* published the most authors listing Global South affiliations (*n =* 3169 authors; 26.4%) followed by *Medical Teacher* (*n =* 1800; 15.0%) and *Advances In Medical Education and Practice* (*n =* 1272; 1.2%). At the article level, nearly all articles (*n =* 373; 99.2%) published in the *African Journal Of Health Professions Education* included at least one author from the Global South followed by *Education for Health* and *Advances In Medical Education and Practice* at 42.5% (*n =* 206) and 33.9% (*n =* 329) respectively. The *GMS Journal for Medical Education* and *Focus on Health Professional Education* included the least number of articles featuring a Global South author; however, both journals are regionally focused (e.g. *GMS Journal for Medical Education* is the official journal of the German Association for Medical Education).

### 3.3 Gender and geographical affiliation

**FIGURE 3:**
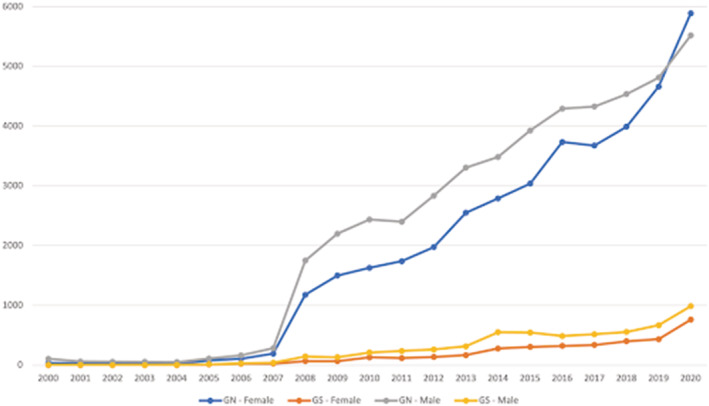
A comparison over time of Global South versus Global North, male and female authors publishing multi‐author articles in medical education journals between 2000 and 2020 [Color figure can be viewed at wileyonlinelibrary.com]

We identified gender and geographical data for 94 922 authors across all authorship positions. Overall females accounted for 42 471 (44.7%) of authorship positions of which 3594 (3.8%) were affiliated with Global South institutions. Males with Global South affiliations represented 6.0% of authors (*n =* 5717) and 10.9% of all males; see Figure 3.

## 4 DISCUSSION

This study, which leverages a unique and sizable data set, describes the author voices present in what has been previously delineated as the medical education literature.^24^ In general, we observed that medical education is a growing field with the large majority of authors affiliated with institutions in the Global North. We also found that women, although much less so for those based in the Global South, are increasingly authoring medical education articles. Taken together, this more complete picture of the medical education literature suggests an imbalance of author voices and further suggests epistemic injustice in medical education science. If diversity of perspectives is important to the field for creativity, innovation and overall excellence in our science,^46^, ^47^ these results indicate that there is considerable room for growth.

Our study intent was to provide the community with an evidence‐based starting point for a conversation or ‘exploratory dialogue’^48^ about epistemic injustice in medical education. Thus, we propose four questions to kickstart those conversations, which we contextualise with our study findings and, where possible, draw on references from medical education and additional fields. We humbly recognise that these four questions are far from exhaustive. What is more, we acknowledge that we do not have all the answers and that our discussion is influenced and limited by our experiences as a predominantly male author team based completely in the Global North.

### 4.1 Question 1: *What knowledge matters in medical education?*

‘Who publishes in leading journals tells students in the Global North and South who counts as an expert, who can produce knowledge and whose ideas matter’.^49^ In light of this claim, our findings suggest that authors from the Global North, and increasingly female authors in those countries, are those whose knowledge seems to matter most. While these findings align with related research^6^, ^7^ and confirm Naidu's description that medical education has a significant ‘northern tilt,’^41^ they raise the question of how as a community we want to define whose and what knowledge matters.

In this study, our bibliometric approach presupposes that it is authors of peer‐reviewed journal articles indexed in the WoS that matter. However, as described in our limitations section below, this approach has shortcomings (e.g. it relies on published literature) and may not align with how the community interprets or wishes that we interpreted whose and what knowledge matters. In answering the question of ‘what matters,’ we encourage the community to think broadly in terms of inclusion of authors in traditional outlets (e.g. including regional journals like *The Asia Pacific Scholar*) and also in terms of emerging publication types and formats. For example, the community could consider what and how resources produced in the Free Open Access Medical Education (FOAM) movement might matter in medical education? FOAM, which is described as a ‘constellation’ of open educational resources (e.g. blogs, videos, podcasts, visual abstracts) authored by medical educators and trainees, has been heavily utilised by trainees and practitioners across the continuum of medical education. And, more recently, it is being factored into promotion and tenure packages and adopted into medical school curricula.^50^

### 4.2 Question 2: *What is the role of the journals*?

Journal publishers, editors and peer reviewers have powerful roles in deciding what is published and thus influencing what is considered knowledge. This suggests that they can and, we propose, should engage in examining and potentially ‘shaking up the structures’^51^ that foster epistemic injustice. Recently, researchers have advocated that those with power in the scholarly publishing system (e.g. publishers, editors, peer reviewers) should exercise ‘responsible agency’.^52^ As described by Jose Medina, responsible agency requires that the individuals in power recognise their position within a system of privilege and oppression.^53^ To this end, several journals have recently undertaken initiatives to act as responsible agents. For example, *Medical Education* has implemented a taskforce that invites readers to engage with the journal; the goal of this effort is to be inclusive of diverse knowledge and perspectives.^54^ Similarly, in 2020, the journal *Teaching and Learning in Medicine* implemented an anti‐racism racism strategy that aims to listen to and amplify the voices of groups minoritized in medical education.^55^ The medical education community might also consider the roles of journals in light of conversations taking place in the literature. For example, the community might discuss: what is a journal's role in ensuring a diverse editorial board? An analysis of 10 medical education editorial boards by Yip and colleagues found a striking lack of diversity with limited representation from lower‐middle and low income countries (LMIC).^56^ We believe that the makeup of editorial boards is potentially important, as research suggests that the inclusion of editorial staff from LMICs is associated with increased publications from those countries.^57^

### 4.3 Question 3: *How can this ‘baseline data’ be used to track the impact of initiatives to move towards epistemic justice?*

Part of our research aim was to provide the community with an evidence base, or a set of baseline data, to start a conversation and begin tracking our progress as a field. However, how these data are used is ultimately up to the medical education community. At a minimum, data such as that presented here could be used to measure if and how there is increased diversity in authorship over time, in terms of gender and geographical location across the field. It could also be used to track authorship diversity within specific journals. However, an important caveat is that we should closely examine a journal's stated aim when considering authorship diversity. For example, *Focus on Health Professions Education* published authors primarily from the Global North, and the *African Journal of Health Professions Education* predominantly published articles by Global South authors. But because these journals are focused on publishing authors from the specific world regions in which they are based, these results make sense and largely align with the journals' stated aims. As such, we would not necessarily expect these author characteristics to change over time. However, similar to Arfeen and colleagues' findings, we observed that the majority of the journals examined here explicitly describe themselves as ‘international’ in focus.^58^ That said, our data align with other studies^5^, ^58^ indicating that most do not at present publish a diverse representation of authors. Additionally, it is important to note that Global South authors may decide not to publish in these journals for reasons beyond (or unrelated to) epistemic injustice. For example, some authors may wish to publish in a regional journal because they want to reach their local audience. In such cases, where a paper is ultimately published, it may be less about epistemic injustice and more about practical considerations.

### 4.4 Question 4: *How can we continue to study epistemic justice in medical education?*

As noted above, studying epistemic injustice is not straightforward.^11^ Yet, we believe this work is imperative. In this study, we used a bibliometric approach, which provides a quantitative view. While valuable, this approach leaves many questions unanswered (e.g. related to author and editor motivations; facilitators and barriers to publishing). More specifically, ‘because counting is never enough’,^59^ we advocate alongside Eva that researchers should seek and implement methods that allow us to understand the context of these data. For example, Connell and colleagues conducted in‐depth interviews with Global South researchers in multiple disciplines. These results gave rich descriptions of the pressure on authors to adopt Global North paradigms when designing and disseminating research. This work also suggested several lessons for how authors ultimately navigated these pressures.^60^ Thus, researchers should consider a variety of methods to better understand this complex issue.

Future research should also examine the intersectionality of author characteristics (e.g. the intersection of an author's gender, national affiliation, race, rank and institutional affiliation). As described by Crenshaw, intersectionality is a ‘lens through which you can see where power comes and collides, where it interlocks and intersects’.^61^ Without this lens, researchers may be unaware of issues and inadvertently commit ‘epistemic violence’ in which marginalised groups are silenced.^62^ For example, in an early interpretation of our data where we examined author characteristics without considering the intersection of their gender and geographic location, it initially appeared that medical education authorship was reaching near gender parity. Although this may be true from an ‘overall numbers’ perspective, this interpretation obscures the fact that only 3.8% of all authors are women based in the Global South. Our initial use of a non‐intersectional frame rendered us unaware of this pronounced disparity, which underscores Crenshaw's caution: ‘if we can't see a problem; we can't fix a problem’.^63^

Our findings should be considered in light of several limitations. First, we used the tool Genderize.io to predict author gender based on first names. However, because gender is a complex social construct that is best determined by the individual, there can be errors with this approach. Additionally, while Genderize.io takes into consideration over 114 million names collected from 195 countries, it is possible that a higher percentage of Asian names were excluded, as these names are a known challenge for gender prediction tools.^64^ Nonetheless, we are unaware of currently available alternatives to establishing author gender in datasets of this scale. Future initiatives might consider encouraging researchers to self‐identify their gender in resources such as ORCID.

Second, we created our sample based on the MEJ‐24, which proposes a seed set of 24 core medical education journals.^24^ While this study expands upon previous efforts by our author team and others to describe a set of core medical education journals,^14^, ^15^ we recognise that the MEJ‐24 sample does not include several relevant medical education articles published in clinical and other specialty‐focused journals (e.g. *JAMA*, *Academic Pediatrics*) or those that are published in ‘mega‐journals’, such as *BMJ Open* or *PLOS One*. Such multidisciplinary journals publish articles across fields.

Third, 22 of the included journals publish articles solely in English, with the exception of the *GMS Journal for Medical Education* and the *Canadian Medical Education Journal*, which simultaneously publishes articles in English and French, respectively. However, there is some representation from national professional associations such as the *African Journal of Health Professions* and *Focus on Health Professions Education*, which is based in Australia. Future work should consider broadening inclusion criteria and recruiting a multilingual team.

Fourth, this study does not include journals specific to other health professions (e.g. dentistry, nursing and pharmacy), although articles from these fields are included in the data set. Future researchers should consider delineating the broader field of health professions education to expand the analysis and better understand this broader context. What is more, our attempt to classify authors in relation to the Global South and Global North by institutional affiliation does not take into account authors that may have shifted locations (i.e. relocated from a Global North to Global South location).

Another important limitation is our reliance on metadata from CrossRef and WoS, which, by definition, represent a subset of the world's literature failing to include many journals that are situated in the Global South.^65^ In addition, while these databases are often used in bibliometric research, they are not infallible and may include data errors that we were unable to detect. Further, in some cases, citations had missing data, especially in regard to institutional and geographic affiliation. In all cases, however, we worked to ‘clean’ our data prior to analysis. The cleaning process required that we create standardised rules. For example, some journals allow authors to list multiple institutional affiliations on the same article. In such instances, which were a minority of cases, we chose to use only the author's first listed institutional affiliation. Furthermore, to make affiliation data manageable, we decided to ‘roll‐up’ institutions that were divided into multiple colleges and institutes to a parent institution. This analytic choice may have slightly inflated publication counts for some parent institutions. Notwithstanding this limitation, and in the interest of transparency and the facilitation of future replications, we have provided details for each rule and how it was applied in our dataset.^31^

## 5 CONCLUSION

Researchers who author medical education articles are the primary drivers of knowledge dissemination and scientific advancement in the field. In this study, we describe the voices of those who have contributed to medical education by examining two decades of the medical education literature, with an emphasis on article and author characteristics. Our findings suggest that medical education scholarship is rapidly growing and evolving, with more female voices being heard, but with a Global North viewpoint that still predominates, which further suggests the possibility of epistemic injustice. Going forward, our community should seek to expand its diversity of voices through evidence‐informed conversations, collaborations and explicit campaigns that solicit scholarly perspectives broadly.

## CONFLICT OF INTEREST

The authors declare no conflicts of interest.

## ETHICAL APPROVAL

Reported as not applicable.

## AUTHOR CONTRIBUTIONS

‘Lauren Maggio, Joseph Costello, Anton Ninkov, Jason Frank and Anthony Artino have all made substantial contributions to the conception and design of the work; the acquisition, analysis and interpretation of data for the work; the drafting and revising of the work; provide final approval of the version to be published; and agree to be accountable for all aspects of the work in ensuring that questions related to the accuracy or integrity of any part of the work are appropriately investigated and resolved’.

## Supporting information




**Appendix S1:** Twenty‐four journals identified^1^

**Table S1:** Top 20 cited authors publishing in 22 medical education journals between 2000–2020
