# Supplementary material for: The voices of medical education scholarship: Describing the published landscape
Source: Med Educ. 2022 Nov 8;57(3):280–9. doi: 10.1111/medu.14959 (PMC10098831; doi:10.1111/medu.14959)
Supplement: Supplementary file 1 — Appendix S1: Twenty‐four journals identified1 [file MEDU-57-280-s002.docx]

**Appendix A: Twenty-four journals identified^1^**

| *Academic Medicine* |
| --- |
| *Advances In Health Sciences Education* |
| *Advances In Medical Education And Practice* |
| *African Journal Of Health Professions Education* |
| *Anatomical Sciences Education* |
| *BMC Medical Education* |
| *BMJ Simulation & Technology Enhanced Learning* |
| *Canadian Medical Education Journal* |
| *Clinical Teacher* |
| *Education For Health* |
| *Focus On Health Professional Education-A Multidisciplinary Journal* |
| *GMS Journal For Medical Education* |
| *International Journal Of Medical Education* |
| *Journal Of Continuing Education In The Health Professions* |
| *Journal Of Educational Evaluation For Health Professions* |
| *Journal Of Graduate Medical Education* |
| *Journal Of Medical Education And Curricular Development* |
| *Journal Of Surgical Education* |
| *Medical Education* |
| *Medical Education Online* |
| *Medical Teacher* |
| *Perspectives On Medical Education* |
| *Simulation In Healthcare-Journal Of The Society For Simulation In Healthcare* |
| *Teaching And Learning In Medicine*  **References**   1. Maggio LA, Ninkov A, Frank JR, Costello JA, Artino AR Jr. Delineating the field of medical education: Bibliometric research approach(es) [published online ahead of print, 2021 Oct 15]. Med Educ. 2021;10.1111/medu.14677. |
