# Supplementary material for: The voices of medical education scholarship: Describing the published landscape
Source: Med Educ. 2022 Nov 8;57(3):280–9. doi: 10.1111/medu.14959 (PMC10098831; doi:10.1111/medu.14959)
Supplement: Supplementary file 2 — Table S1: Top 20 cited authors publishing in 22 medical education journals between 2000–2020 [file MEDU-57-280-s001.docx]

Supplemental Table 1: Top 20 cited authors publishing in 22 medical education journals between 2000-2020

| **Author (gender, country)** | **Total citations** | **# Articles published since 2000** | **# Citations for most cited article** | **Average citation rate*** |
| --- | --- | --- | --- | --- |
| Cees van der Vleuten (male, Netherlands) | 15733 | 407 | 658 | 38.7 |
| Olle ten Cate (male, Netherlands) | 8697 | 215 | 561 | 40.5 |
| Geoffrey Norman (male, Canada) | 8514 | 209 | 1435 | 40.7 |
| David Cook (male, United States) | 7887 | 114 | 1661 | 69.2 |
| Albert Scherpbier (male, Netherlands) | 7149 | 228 | 337 | 31.4 |
| Kevin Eva (male, Canada) | 7106 | 176 | 568 | 40.4 |
| William McGaghie (male, United States) | 6993 | 101 | 1845 | 69.2 |
| Glenn Regehr (male, Canada) | 6146 | 126 | 568 | 48.8 |
| Eric Holmboe (male, United States) | 5756 | 155 | 384 | 37.1 |
| Karen Mann (female, Canada) | 5326 | 68 | 935 | 78.3 |
| Lambert Schuwirth (male, Australia) | 5220 | 135 | 935 | 38.7 |
| Steve Durning (male, United States) | 5174 | 219 | 658 | 23.6 |
| Mohammadreza Hojat (male, United States) | 5146 | 68 | 291 | 75.7 |
| Barry Issenberg (male, United States) | 5026 | 43 | 751 | 116.9 |
| Lorelei Lingard (female, Canada) | 4540 | 141 | 1845 | 32.2 |
| Liselotte Dyrbye (female, United States) | 4432 | 53 | 271 | 83.6 |
| Tait Shanafelt (male, United States) | 4184 | 36 | 1235 | 116.2 |
| Yvonne Steinert (female, Canada) | 4174 | 103 | 1235 | 40.5 |
| Mohsen Tavakol (male, United Kingdom) | 4057 | 34 | 690 | 119.3 |
| Reg Dennick (male, United Kingdom) | 4000 | 33 | 3310 | 121.2 |

*Average Citation Rate = total citations/total articles published. Note that citations are based on a raw count of citations, which may be impacted by time.
